# Feasibility of a T-Shirt-Type Wearable Electrocardiography Monitor for Detection of Covert Atrial Fibrillation in Young Healthy Adults

**DOI:** 10.1038/s41598-019-48267-1

**Published:** 2019-08-13

**Authors:** Nobuaki Fukuma, Eriko Hasumi, Katsuhito Fujiu, Kayo Waki, Tsuguyoshi Toyooka, Issei Komuro, Kazuhiko Ohe

**Affiliations:** 1 Department of Cardiovascular Medicine, Graduate School of Medicine, The University of Tokyo, Tokyo, Japan; 2 Department of Ubiquitous Health Informatics, Graduate School of Medicine, The University of Tokyo, Tokyo, Japan; 3 Department of Advanced Cardiology, Graduate School of Medicine, The University of Tokyo, Tokyo, Japan; 4 Business Development of Healthcare Business Smart-Life Solutions Department, NTT DOCOMO, INC., Tokyo, Japan; 5 Department of Biomedical Informatics, Graduate School of Medicine, The University of Tokyo, Tokyo, Japan

**Keywords:** Cardiac device therapy, Electrodiagnosis

## Abstract

Covert atrial fibrillation (AF) accounts for cryptogenic stroke aetiology in elderly patients and in younger populations. However, asymptomatic AF is difficult to diagnose based on a short electrocardiography (ECG) recording. We evaluated the feasibility of a self-applied continuous ECG monitoring device that can record automatically, easily, and noninvasively in a younger population. We investigated community screening for asymptomatic AF using a wireless single-lead ECG with an electrode embedded in a T-shirt. One hundred men with a CHADS2 score ≥1 who were free from AF and <65 years of age were enrolled. We instructed the participants to wear ECG monitoring devices for at least 4 days/week over 2 months. The proportion of participants with newly detected AF (NDAF) and the monitoring time were evaluated. The mean CHADS2 score was 1.43 ± 0.62. The mean patient age was 52.5 ± 5.4 years. The mean monitoring time was 222 ± 199 hours. NDAF continuing for >30 seconds was detected in 10 participants (10.0%). AF continuing for >6 minutes was detected in 2 participants (2.0%). The T-shirt-type wearable ECG monitoring system was suitable for continuous, daily long-term use among young people with high physical activity, and it had the distinct capability of identifying covert AF.

## Introduction

Atrial fibrillation (AF) causes cardiogenic stroke, accounting for approximately one-third of all brain infarcts^1,2^. Since the proportion of asymptomatic AF within all AF episodes is almost 69.0%^3^, the detection of asymptomatic AF can be difficult in general. However, even intermittent asymptomatic AF episodes substantially increase the risk for ischemic stroke and systemic embolism^4^. Although up to 20% of patients with ischemic stroke have a previous diagnosis of AF, 7.7% of patients with stroke were diagnosed with the first-detected AF on the day of stroke onset, and 23.7–30% of patients were diagnosed after stroke attack^5,6^. Thus, the earlier detection of asymptomatic AF and administration of anticoagulation therapy before stroke attack are desired to prevent disability in patients at high risk for cardiogenic stroke.

Current guidelines suggest performing 24 or more hours of ECG monitoring to rule out AF in patients with ischemic stroke^7,8^, unless another cause for stroke is apparent. However, it was reported that the detection rate of conventional strategies including in-hospital monitoring, serial ECG monitoring, Holter monitoring, and monitoring using an external event or loop recorders is low^4^ due to the short monitoring period. Implantable cardiac monitoring (ICM) is useful for detecting intermittent asymptomatic AF in patients with embolic stroke of an undetermined source. A randomised controlled trial showed that long-term monitoring with an ICM was more effective than conventional follow-up for detecting AF in patients with cryptogenic stroke^5^. However, ICM is not indicated for screening of individuals with symptomatic AF as primary prevention of stroke^9^. Recently, noninvasive long-term ECG monitoring has been a focus in the development of a screening method for undiagnosed AF. Wearable continuous ECG monitoring patches and iPhone-based lead-I ECG (iECG) were found to be superior to conventional follow-up for detecting AF in randomised controlled trials^10–12^. However, these studies have been conducted in only elderly patient populations. The proportion of first-ever strokes in young adults comprises 5–20% of all strokes^13,14^, and AF is a major cause of stroke in young adults^15^. Therefore, the detection of covert AF is as necessary for first stroke prevention in young adults as it is in elderly people.

We evaluated the feasibility of a noninvasive, wearable ECG monitoring with an electrode embedded in a T-shirt that is suitable for young people’s lifestyle and sports activities.

## Methods

### Participant population

**Figure 1:**
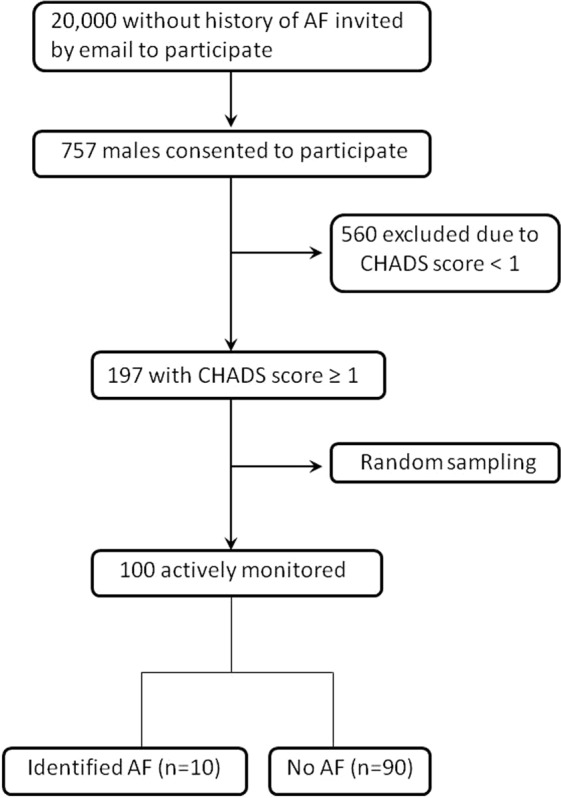
Recruitment of local participants <65 years of age with a CHADS2 score ≥1. AF, atrial fibrillation.

We contacted 20,000 employees of NTT DOCOMO, INC. (Tokyo, Japan) for screening by using an intranet-based e-mailing system. All the employees were <65 years of age and had never been diagnosed with AF during biannual ECG examinations. Overall, 757 men consented to participate, but 560 were excluded because their CHADS2 score was <1. A random sample of 100 volunteers was chosen from the 197 volunteers with a CHADS2 score ≥1 (Fig. 1). We calculated the CHADS2 score for each participant by using medical history data obtained through both an interview and medical record review after participants’ consent was obtained.

### Ethical statements

This study was approved by the institutional ethical committee of the University of Tokyo (approval number: 11125), and written informed consent was obtained from all participants in this study. All aspects of the study conformed to the guidelines of the 2013 WMA Declaration of Helsinki.

### Screening for the AF procedure

**Figure 2:**
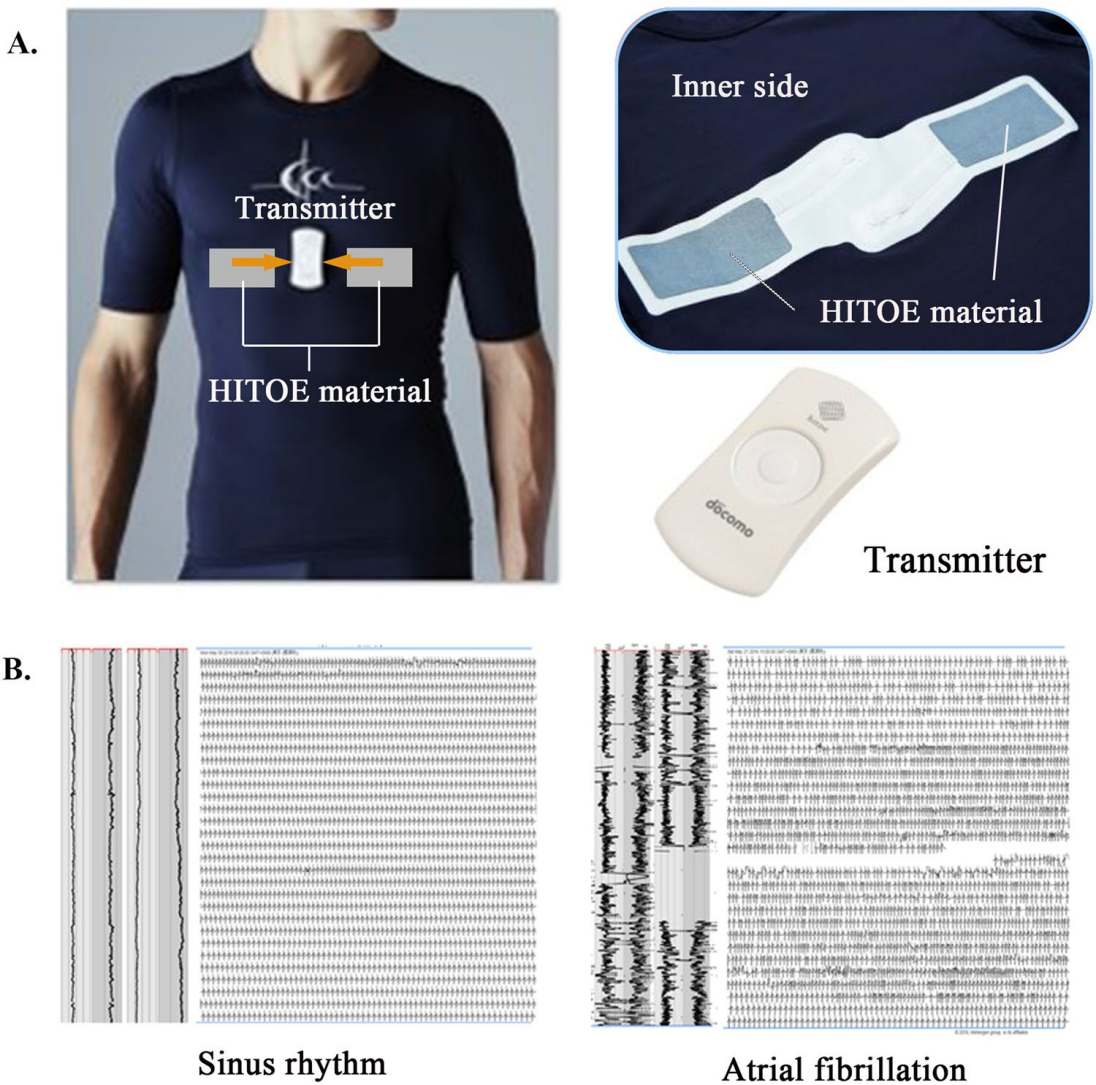
The long-term monitoring system with Hitoe material. (**A**) The system comprises a T-shirt made of Hitoe material, which is highly electrically conductive and flexible and embedded on the inside, and the transmitter is placed on the midline of the chest. (**B**) Recorded electrocardiography (ECG) waveform, sinus rhythm, and paroxysmal atrial fibrillation (AF) by ECG monitoring system with Hitoe material.

Participants were instructed to wear a T-shirt for at least 40 hours a week (4 days a week for 10 hours each time with a target of 320 hours) over 2 months. In this study, we used a wearable ECG that is a wireless single-lead device with an electrode embedded into a T-shirt for community screening to identify AF. The device is composed of a T-shirt embedded with a highly electrically conductive material (Hitoe, NTT DOCOMO Inc., Tokyo, Japan) that functions in the same manner as the electrolyte patches and Hitoe transmitter 01 (NTT DOCOMO Inc.). The Hitoe is a woven fabric created by silk-coated fibre surfaces with an electrically conductive polymer material (PEDOT-PSS: poly (3,4-ethylene dioxythiophene): poly (styrene sulfonate))^16^ (Fig. 2A). The T-shirt (C3fit IN-pulse, GOLDWIN Inc., Tokyo, Japan) is made of a stretchable, form-fitting fabric that is designed for sports. Participants chose the size of the T-shirt (medium or large) according to their body size. Because this system is washable, participants can wash and dry this shirt anytime, and the shirt can be worn repeatedly. The Hitoe electrodes are located on the right and left of the sternum, and the position of the Hitoe electrodes is comparable to bipolar chest leads, CC5. The participants were free to wash their T-shirt if they did not wear it. ECG data were sent from the Hitoe transmitter 01 to the participants’ smartphones by Bluetooth, and then their smartphones sent ECG data to our data server on the 4G network (Fig. 2B). The transmitter (size, 70 mm × 37 mm × 9.6 mm; weight, 24 g) was attached on the centre position of the left and right Hitoe materials. ECG noise was removed using a band-pass filter between 1 and 40 Hz in the smart phone application developed by NTT DOCOMO, Inc. Cardiologists analysed all ECG waveforms manually and diagnosed newly detected AF (NDAF). Proper treatments were provided when NDAF was detected.

### Outcomes

We investigated the feasibility and acceptability of the wearable ECG monitoring system. The detection of NDAF was also evaluated. AF was defined as a 30-second ECG recording of an irregular rhythm without a P wave^17^. The AF identification rate and monitoring duration were calculated. All statistical analyses were performed using SPSS software, version 23.0 (SPSS Inc., Chicago, IL).

## Results

All 100 participants underwent 60 days of follow-up. The mean participant age was 52.5 ± 5.4 years (range, 37 to 63 years). The mean CHADS2 score was 1.43 ± 0.62, and the mean CHA2DS2-VASc score was 1.53 ± 0.72 (Table 1). The average monitoring time was 224.7 ± 199.0 hours (range, 0.05 to 945 hours). Thirty participants (30%) achieved the target total wearing time of 320 hours. The wearing times of 2 individuals were less than 24 hours because they reported feeling uncomfortable; One extremely obese participant complained that the T-shirt was too small for his body size, and the other participant noted discomfort with the Hitoe material, which absorbed sweat. Consequently, the patient acceptance rate for Hitoe was 98% (Table 2). The wearing time and recording time were almost the same, and recording failure due to noise was <1%.

**Table 1:** Baseline Characteristics.

Variable	Overall (n = 100)
Age, mean, y	52.5 ± 5.4
<65 years	100 (100%)
Male	100 (100%)
Comorbidities	
Diabetic mellitus	36 (36%)
Hypertension	88 (88%)
Heart failure	1 (1%)
Previous stroke	9 (9%)
Vascular disease	10 (10%)
CHADS2 score, mean	1.43 ± 0.62
1	64 (64%)
2	29 (29%)
3	7 (7%)
4	0 (0%)
CHA2DS2-VASc score, mean	1.53 ± 0.72
1	59 (59%)
2	30 (30%)
3	10 (10%)
4	1 (1%)

**Table 2:** Results of ECG monitoring data.

	Overall (n = 100)
Monitoring duration (hours)	222.4 ± 199.3
Acceptance	98 (98%)

**Figure 3:**
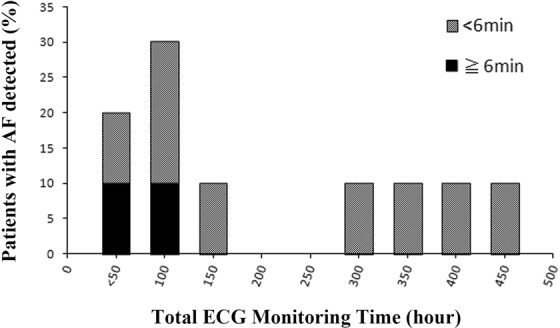
Percentage frequency distribution of total electrocardiography monitoring time in patients with newly detected atrial fibrillation.

AF was detected in 10 participants (10.2%). Among them, 2 participants (2%) had AF lasting ≥6 minutes at least once or >2 months (Table 3). All individuals in this study had at least 1 stroke risk factor. We also investigated the incidence of NDAF lasting <6 minutes and the incidence of AF lasting >6 minutes in groups of patients with an intermediate (CHADS2 score of 1), high (CHADS2 score of 2), and very high stroke risk (CHADS2 score of ≥3). All incidences of NDAF (both ≥ 6 minutes and <6 minutes) were observed in patients with an intermediate level of stroke risk factors (100% of these instances; Supplemental Fig. 1). Since 2 patients with NDAF lasting ≥6 minutes had no symptoms, the wearable monitoring device could identify asymptomatic AF. However, the total ECG monitoring time in both patients was a relatively short time of <100 hours, namely, 31 hours for 1 case and 93 hours for the other (Fig. 3). We investigated the NDAF in the recorded ECG, which revealed that this duration was 7.5 hours for 1 case and 12 minutes for the other (Supplemental Fig. 2).

**Table 3:** Detection rate of Atrial Fibrillation.

	Overall (n = 98)
Newly detection of AF $\geqq$30 sec	10 (10.2%)
Newly detection of AF $\geqq$6 min	2 (2.0%)

AF, atrial fibrillation.

## Discussion

AF-related strokes commonly result in a greater disability than ischemic stroke secondary to arterial disease^18^, and AF-related strokes often have devastating consequences on quality of life and workability. Some studies have indicated that the incidence of stroke in young adults has increased, and the long-term mortality after stroke in young adults is higher than expected^19^. In addition, from an economic aspect, stroke in young adults has large long-term socioeconomic impacts, compared with stroke in older adults. Therefore, we tried to measure NDAF as a major cause of stroke by long-term monitoring ECG with Hitoe, which is suitable for the highly active lifestyle of young adults. Recently, some studies reported that undiagnosed AF was identified using a remote ECG monitoring system. NDAF was identified in 3.8% of the population older than 65 years by twice-weekly recordings and transmission of a 30-second single-lead iECG (Alive Cor^®^ Heart Monitor; Alive Cor, Inc., USA) over 12 months^12^, and in 3% of the participants older than 75 years of age by twice-daily intermittent single-lead ECG (Zenicor)^20^. Recent reports indicated that immediate monitoring with home-based wearable ECG sensor patches (iRhythm Zio ^XT^) resulted in a high AF diagnosis rate of 3.9% after 4 months in elderly individuals (average age, 72.4 years) with a high risk of stroke^10^. In the current study, the AF (≥6 minutes) detection rate was 2.0% (detection rate of AF < 6 minutes was 10.2%) over 2 months. These results were close to the detection rate reported in other remote ECG device monitoring trials^10,12,20^.

The T-shirt-type ECG monitoring system is made of stretchable fabric that is comfortable to wear and washable. Therefore, the monitoring system with Hitoe can be applied in many situations, including sports, leisure, and work. The Hitoe material could clearly record the ECG waveform as well as other wearable ECG sensors with electrode patches because Hitoe was in close contact with participants’ skin. Baseline drift in ECG signal and discontinuities of ECG recording occurred in circumstances where participants leaned far forward, causing Hitoe to be separated from the skin. However, these instances occurred over a short time and had no significant effect on the analysis of ECG. In contrast to ECG electrode patches, the Hitoe material prevented skin erosion. The highly conductive fibers of Hitoe permitted this remote monitoring system to record the ECG waveform clear enough to identify AF without invasion of the skin. These factors lead to the high detection rate of AF in this study. Hitoe is flexible because it can record during exercise and detect an arrhythmia, such as long QT syndrome, in patients with syncope.

In this study, only 30% of participants wore the device for much less than the targeted duration. The main reason why many participants’ recorded ECG monitoring was for a short time is that asymptomatic participants had difficulty participating in active monitoring. In the mSToPS randomised clinical trial, Steinhubl *et al*. reported that a self-applied wearable ECG patch resulted in a higher rate of AF diagnosis. However, a substantial number (38%) of individuals who were initially interested in participating changed their minds and never wore the patch^10^. Based on this report, it is difficult for asymptomatic participants to start monitoring and participate in continuous active monitoring. We believe that one method for resolving this problem is to keep close contact with the participants. In their randomised clinical trial, Kitzman *et al*. indicated that caloric restriction or aerobic exercise training improves exercise capacity and quality of live in obese older patients with heart failure with preserved ejection fraction. Of note, the participants received telephone calls every 2 weeks from staff as a reminder call and monitored all the food they consumed weekly. Frequent contact with participants resulted in high exercise attendance (84%) and diet adherence (99%)^21^. Therefore, it is thought that contact via regular telephone calls from the physician and staff or medical alert system of the smart phone application in active ECG monitoring encourage participants to wear the ECG monitoring system for the target wearing time.

### Study limitations

This study is a descriptive study, not a randomised controlled trial. The overall patient population was small and had a sex bias. Although women account for 22% of all employees, all participants were men, and no woman consented to participant. Additionally, this study used short monitoring durations of <10 hours in 8% of participants and <50 hours in 24% (Supplemental Fig. 3). Some participants complained about wearing the Hitoe material because the high hydrophilicity of the inner side of the T-shirt caused it to become soaked with sweat. However, this issue with the shirts has been ameliorated, and new revised shirts have been released. Future improvements of compliance in terms of comfortableness are expected. Additionally, further studies need to include a larger sample size and assess the measures herein for a longer duration. The standard ECG recording system was not used to determine whether the wave recorded by Hitoe is really arrhythmia or not.

## Conclusions

The T-shirt made with Hitoe material containing the long-term monitoring system detected AF in 10% of young adult participants without a history of AF; furthermore, AF lasted for >6 minutes in 2% of the participants. This wearable ECG monitor had AF-detection capability similar to that of other wearable devices. Moreover, this system does not use an electrode patch, thus avoiding skin damage. Based on these points, this monitoring system can play an important role in the detection of covert AF and the prevention of cardiogenic stroke in younger populations.

## Supplementary information

LaTeX Supplementary File

## Data Availability

The raw data are available on request from corresponding authors.
