# Supplementary material for: Feasibility of a T-Shirt-Type Wearable Electrocardiography Monitor for Detection of Covert Atrial Fibrillation in Young Healthy Adults
Source: Sci Rep. 2019 Aug 13;9:11768. doi: 10.1038/s41598-019-48267-1 (PMC6692346; doi:10.1038/s41598-019-48267-1)
Supplement: Supplementary file 1 — LaTeX Supplementary File [file 41598_2019_48267_MOESM1_ESM.pdf]

Feasibility of a T-Shirt-Type Wearable Electrocardiography Monitor for Detection of Covert Atrial Fibrillation in Young Healthy Adults

Nobuaki Fukuma[1*], Eriko Hasumi[1,2*,**], Katsuhito Fujiu[1,2,3**], Kayo Waki[2], Tsuguyoshi Toyooka[2,4], Issei Komuro[1], Kazuhiko Ohe[5]

Supplemental figure 1.

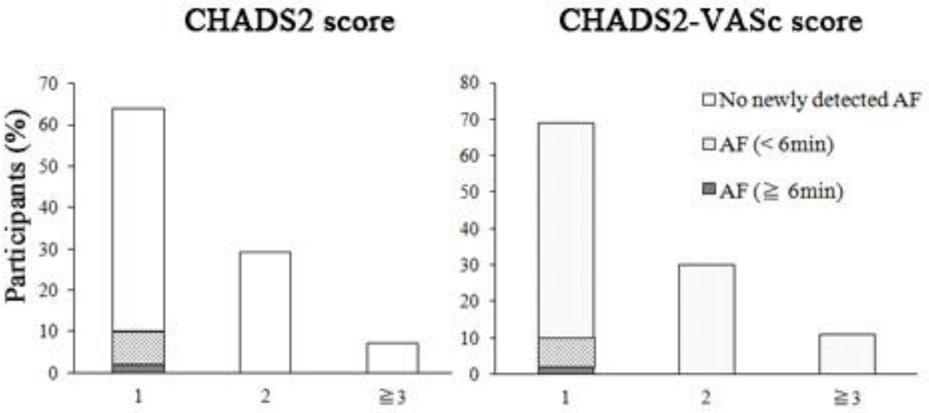

Percentage frequency distribution of CHADS2/CHA2DS2-VASc scores in participants with no newly detected atrial fibrillation (AF), newly detected AF (NDAF) (<6 minutes), and NDAF (≥6 minutes).

Supplemental figure 2.

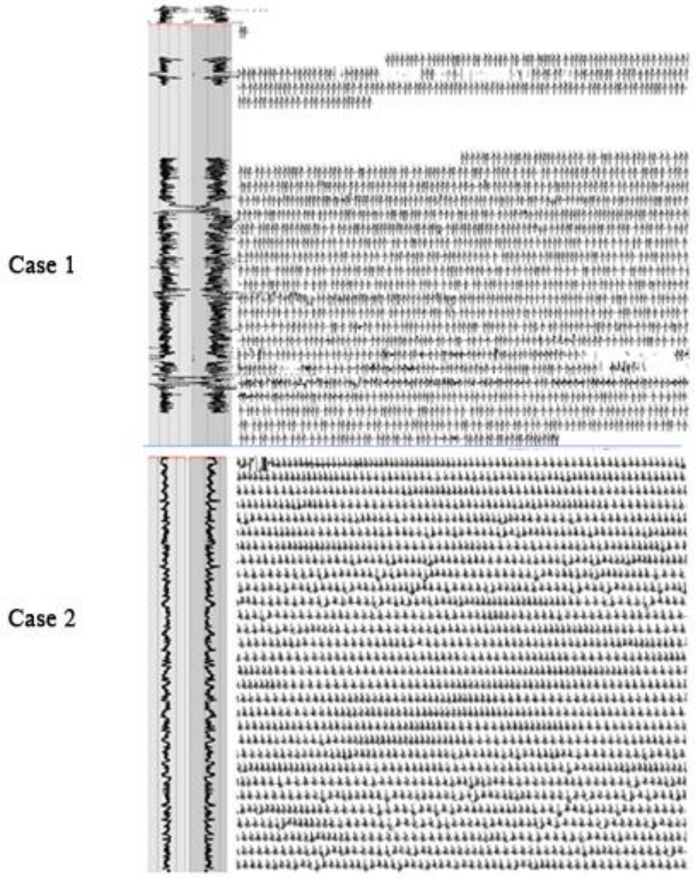

Electrocardiographies in the 2 patients with newly detected atrial fibrillation (NDAF) lasting ≥6 minutes. In case 1, NDAF lasted 7.5 hours, and in case 2, NDAF lasted 12 minutes.

Supplemental figure 3.

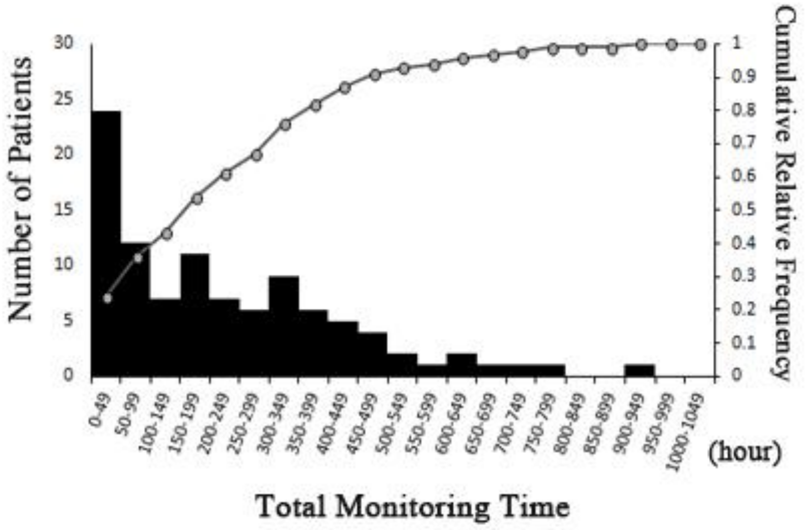

Total Electrocardiography monitoring time and cumulative time over 2 months in young adults.
